# Information-Based Medicine in Glioma Patients: A Clinical Perspective

**DOI:** 10.1155/2018/8572058

**Published:** 2018-06-13

**Authors:** Joeky Tamba Senders, Maya Harary, Brittany Morgan Stopa, Patrick Staples, Marike Lianne Daphne Broekman, Timothy Richard Smith, William Brian Gormley, Omar Arnaout

**Affiliations:** ^1^Computational Neuroscience Outcomes Center, Department of Neurosurgery, Brigham and Women's Hospital, Harvard Medical School, Boston, MA, USA; ^2^Department of Neurosurgery, University Medical Center Utrecht, Utrecht, Netherlands; ^3^Department of Biostatistics, Harvard T.H. Chan School of Public Health, Harvard University, Boston, MA, USA; ^4^Department of Neurosurgery, Leiden University Medical Center, Leiden, Netherlands

## Abstract

Glioma constitutes the most common type of primary brain tumor with a dismal survival, often measured in terms of months or years. The thin line between treatment effectiveness and patient harm underpins the importance of tailoring clinical management to the individual patient. Randomized trials have laid the foundation for many neuro-oncological guidelines. Despite this, their findings focus on group-level estimates. Given our current tools, we are limited in our ability to guide patients on what therapy is best for them as individuals, or even how long they should expect to survive. Machine learning, however, promises to provide the analytical support for personalizing treatment decisions, and deep learning allows clinicians to unlock insight from the vast amount of unstructured data that is collected on glioma patients. Although these novel techniques have achieved astonishing results across a variety of clinical applications, significant hurdles remain associated with the implementation of them in clinical practice. Future challenges include the assembly of well-curated cross-institutional datasets, improvement of the interpretability of machine learning models, and balancing novel evidence-based decision-making with the associated liability of automated inference. Although artificial intelligence already exceeds clinical expertise in a variety of applications, clinicians remain responsible for interpreting the implications of, and acting upon, each prediction.

## 1. Review

Glioma constitutes the most common type of primary malignant brain tumor with an incidence of over 20,000 new cases a year in the United States[1]. Treatment strategies significantly improved over the last decades and include maximal safe resection, temozolomide chemotherapy, radiotherapy, and immunotherapy [2]. Gliomas are subgrouped into low grade gliomas (LGG) and high-grade gliomas (HGG) which include glioblastoma, on the basis of tumor genetic and molecular markers [3]. However, they are noncurative, due to the aggressive nature of the tumor. The current median expected survival after diagnosis in glioma patients remains, therefore, solely a few months for HGGs, and years for LGGs, despite optimal treatment [1]. Furthermore, significant morbidity is associated with both the disease condition and its therapeutic solutions [4].

## 2. Classical Statistics, Machine Learning, and Deep Learning

For much of the history of scientific inquiry, classical frequentist statistics have formed the basis of data analysis, with randomized clinical trial (RCT) design forming the pinnacle of evidence-based medicine [5]. There are presently 270 open and enrolling clinical trials for glioblastoma, which employ a range of treatment modalities including investigational drugs, biologics, standard-of-care treatment, radiation, and surgical intervention to try to find an optimal cure for glioblastoma. Recruitment into clinical trials is largely based on a histological diagnosis of glioma, with or without specific genetic markers such as isocitrate dehydrogenase (IDH) and O6-methylguanine-DNA methyltransferase (MGMT) gene methylation. Despite the huge advances in glioma management that have resulted from these and prior RCTs, the frequentist data analysis methods employed limit the ability to draw conclusion about the efficacy of treatment in individual patients, based on their unique characteristics.

**Figure 1:**
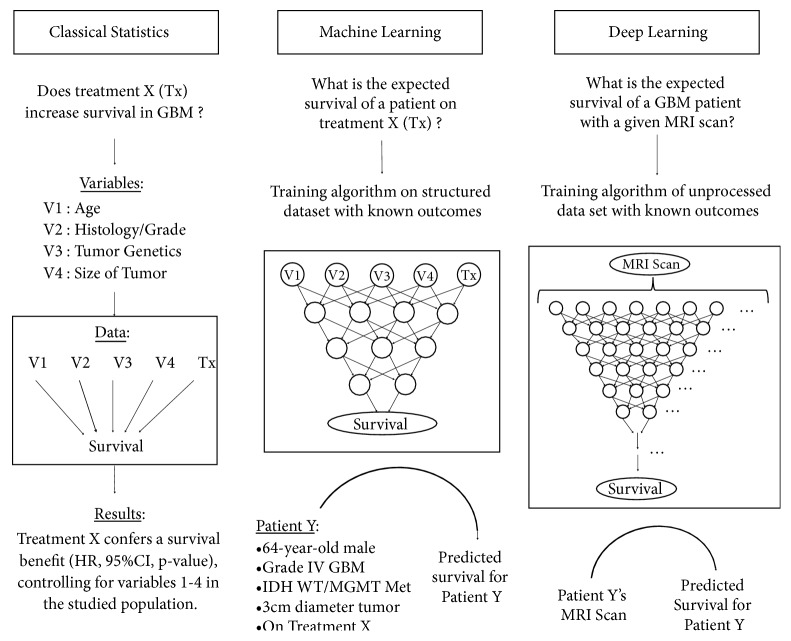
Classical statistics, machine learning, and deep learning approaches as can be applied to the care of GBM patients.

Classical statistical methods were developed to evaluate the strength of association or effect of covariates (e.g., treatment) and a single dependent variable (e.g., survival) within a sample population, with the aim of generalizing these conclusions to the larger population (**Figure 1**) [6]. In that sense, the results of trials and cohort studies remain averaged estimates based on the total study cohort but do not necessarily apply to the same extent to individual patients. For example, regression analysis can quantify the association between chemotherapy and survival into a single coefficient, thereby providing an understanding of its effect size; however, the actual effect size remains different for each individual patient. By design, classical statistical methods, such as regression, make various assumptions about the relationships within the data to make inferences about a larger population. For this reason, these methods are limited in their ability to assess the interactions between covariates and analyze complex nonlinear relationships with the outcome. These limitations make traditional statistical algorithms less well suited to model real-life complex systems and for applications in personalized clinical care in the era of Big Data [7].

The use of machine learning provides clinicians with the analytical support for personalizing treatment decisions. Machine learning is the branch of artificial intelligence that gives computer algorithms the ability to learn through experience rather than the explicit programming of a set of rules. Statistical models typically describe the relationship between clinical factors and the outcome, whereas machine learning models typically seek to predict the outcome using these factors as input features. In the context of glioma patients, statistical approaches are particularly well suited for identifying treatment strategies and other factors associated with survival, whereas machine learning uses these factors to predict survival. Although similar mathematical models are used in both fields, modern machine learning algorithms prioritize prediction over inference, even if it is achieved at the cost of its interpretability [8]. This allows machine learning algorithms to model complex structure within the data, even those that are potentially undetectable or not even understandable by humans, in order to achieve the highest discoverable prediction performance. Characterizing the interaction between a variety of features to pursue the highest prediction performance has been, for example, of particular interest in genomic research on glioma patients. The association of a single gene and the outcome is highly dependent on the rest of the genetic profile. Machine learning algorithms have demonstrated great performance in modeling interactions and subtle patterns within the genetic profile [9, 10].

The application of traditional machine learning is limited, however, to structured data. Typically, unstructured data from diagnostic modalities is summarized in tabular format to make the data more suitable for analysis. In the context of imaging analysis in glioma patients, this means that a neuroradiologist has to manually score predetermined variables, such as tumor location, size, extension, and contrast-enhancement pattern. However, this simplification reduces the amount of information that is contained in the raw data, places a significant time burden on the clinician or researcher, introduces human subjectivity with regard to the generation and selection of input features, and limits this selection to features that can only be measured by humans. As we are entering an era of Big Data, more and more patient-generated health data on glioma patients is heterogeneous in format, such as imaging scans, pathology slices, monitoring devices, and free text clinical communications [11].

Deep learning has emerged as a family of techniques to maximize our use of all of these data streams and has demonstrated new and astonishing results in a variety of fields [12]. Today, deep learning implies the fitting of an artificial neural network. Artificial neural networks are inspired by the neural network of the brain and organized in hierarchical layers of interconnected nodes. By stacking many network layers, a deep learning model may consist of millions of parameters, each of which can be jointly optimized by training on labeled data for prediction [13]. Instead of a neuroradiologist that has to manually score the MRI scan according to basic variables, deep learning algorithms can ingest the raw MRI scan considering each voxel as individual input feature, identify meaningful representations within the data, and drop information that is not contributing. For example, nodes in the lower layers of a deep learning model for computer vision might be susceptible for simple straight lines, hidden layers can learn how to detect shapes by recognizing combinations of activations in the lower layers, and the top layers use this condensed knowledge to produce clinically meaningful estimates, such as for diagnostic classification, volumetric segmentation, and outcome prediction. This process of condensing raw data to meaningful features within the model is called feature extraction and allows raw data to speak for itself [12].

## 3. Clinical Application

The wide use of complex diagnostic and therapeutic modalities in neuro-oncology calls for automated methods to analyze this data, but it also provides an opportune framework for the development of machine learning models. The utility of machine learning in neuro-oncology has, therefore, been explored extensively and showed excellent results, at times even beyond the performance of clinical experts [14]. Traditional machine learning methods have been widely explored to develop diagnostic and prognostic prediction tools based on structured data, but also as a final classifier for complex data after feature extraction. For example, random forest and support vector machines have been demonstrated to accurately predict survival in glioma patients based on basic clinical features alone [15] or features extracted from MRI and functional imaging [16–19].

Deep learning is particularly well suited to enhance the analysis of medical imaging data because the subtle but relevant patterns within the digital information are not always detectable by clinical experts [20]. Informing clinical decision-making with insight extracted from medical imaging data can have a pivotal impact in the care of glioma patients. Clinical management in glioma patients is highly dependent on the histopathological subtype, tumor grade, genetic profile, and spread of the tumor. Radiographic scoring criteria, such as the Response Assessment in Neuro-Oncology (RANO) criteria, have been developed to improve the accuracy and consistency of radiological assessment by neuroradiologists; however, the predictive performance of these scoring criteria is limited. Pathological assessment remains, therefore, the cornerstone of tumor characterization, which drives clinical management towards surgery and exposes patients to the perioperative risks associated with highly invasive neurosurgical procedures.

Deep learning models trained on medical imaging data achieve high accuracy with regard to histopathological diagnosis [21], genetic profile [22], tumor grade [23], and prognosis [24], thereby enabling clinicians to derive insight into tumor characteristics and anticipate expected outcomes of glioma patients prior to any invasive diagnostic procedures. This can be practice changing for patients suspected of disease progression or malignant transformation, especially when operative treatment is nonpreferential or even unnecessary due to absence of the disease. Furthermore, deep learning is capable of providing automated segmentations of the tumor [25], critical brain structures [26], and even peritumoral infiltration that is not yet visible on the scan [27]. These segmentations help neurosurgeons to target surgical resection to areas at risk for infiltration and recurrence while preserving functional tissue. Improving the extent of resection leads to fewer subsequent surgeries, better response to adjuvant therapy, longer progression-free and overall survival, and improved quality of life [28]. Integrating radiological information with clinical, genomic, and histopathological data facilitates the construction of an individualized disease phenotype, thereby providing an understanding of tumor progression, survival, and treatment effectiveness [29].

## 4. Future Challenges

Given the unprecedented variety and accuracy of machine learning algorithms in answering specific types of clinical questions, the use of these methods in healthcare will transform clinical care [30]. Machine learning provides very powerful tools to solve critical problems with complex data; however, it is not necessarily the best approach for all computational and clinical problems. Regression analysis and other methods for statistical inference will remain central for the implementation of novel treatment strategies by clarifying effectiveness on group-level, while machine learning models will later shift our understanding of treatment effectiveness from group-level to patient-level estimates. Because this shift from group-level to patient-level estimates requires collection of sufficient training data, high predictive performance might not be achieved until after the novel treatment strategy has been implemented in clinical care.

### 4.1. Assembly of Training Datasets

Despite their significant promise, some hurdles remain associated with the implementation of machine learning in clinical practice. Unlike the data sources used in most commercial applications of machine learning, health information including diagnostic imaging, genetic testing, and pathology specimens is protected under the law. In the United States, this is regulated by the Health Insurance Portability and Accountability Act of 1996 (HIPAA) [31]. Research related access to private health information requires approval through an institutional review board. While these regulations are crucial to maintain patient privacy, they limit the sharing of data with collaborators at other institutions. As a result, research endeavors are often limited to single-institutional data, which negatively impacts the performance and generalizability of the resulting model. One way to overcome this barrier is through the use of deidentified databases, such as the national Surveillance, Epidemiology and End Results (SEER) program that contains deidentified information about oncologic patients [32]. Though a potentially powerful and large-scale data resource, these population-based databases have limited practical utility as the included data are often limited to basic clinical features.

The volume and complexity of the unstructured data, such as MRI, genomics, and free text clinical notes, hamper the assembly of multicenter, or even nationwide data sets. These types of datasets are still in their infancy and will require a more rigorous organizational backing, such as systematizing the inclusion criteria of studies and providing quality assurance of both image quality and veracity of associated clinical data [33]. Variation in image acquisition methods poses a barrier to image-based modeling across institutions. Imaging results acquired on different scanners may not be directly comparable. Similarly, the exact acquisition parameters for a given MR sequence (e.g., contrast-enhanced T1-weighted axial image) may vary within and between institutions [34]. Besides the technical hurdles, the cross-institutional aggregation of complex unstructured data is complicated by privacy issues as well. Although images can be deidentified and defaced (modified so that the patient's face cannot be reconstructed from the cross-sectional images slices) prior to submission, these deidentification and encryption methods may still fail because the data remains so rich in personal detail. Even if anonymization systems are put in place, combining multiple data points might still reveal an individuals' identity. Currently, most models developed in the scientific realm have been trained on well-curated institutional datasets. It is yet unknown how well models trained on local research data generalizes to real-world clinical data.

### 4.2. Validation of Models and Acceptance by Clinicians

Machine learning algorithms are typically left unequipped with explanations that accompany their predictions, which conflicts with the fundamental inquisitive human nature that drives scientific inquiry [24]. However, artificial intelligence technology holds the potential of producing accurate and individualized prediction. Nevertheless, clinicians may remain uncomfortable trusting clinical decision-making to such “black-box” algorithms for which the underlying mechanisms are not fully understood. This tradeoff is not a new concept in medicine; many pharmaceutical drugs are approved based on the simple observation that they are safe and effective, yet often lacking a complete understanding of the therapeutic mechanism [35]. The key difference is that the safety and efficacy of machine learning algorithms are dynamic and dependent on multiple factors including the quality of the data on which these algorithms are trained, evaluated, and utilized. These factors are, however, highly subject to change. A sudden and undetected change in patient population or data acquisition reduces model performance, and a delay in detecting these deviating performance trends can result in detrimental patient outcomes. For this reason, occasional revalidation of these models may prove necessary in a way that is not for drugs and medical devices (i.e., once they are approved for use, drug or device efficacy does not necessarily need to be reexamined unless new adverse events are reported in stage IV trials). Furthermore, the dynamic performance and lack of interpretability of advanced prediction models also pose challenges regarding liability when these models serve as decision-support tools for clinicians [36].

As machine learning algorithms are trained on retrospective data, these algorithms may also mirror human biases in clinical decision-making [37]. For example, including race or ethnicity in a clinical prediction model may contribute to its performance because race can, to a degree, be an approximate of biogenetic constitution; however, it may also be associated with socioeconomic status, which may cause issues of health equity and care accessibility to affect model prediction in unforeseeable ways. As machine learning algorithms learn from existing patterns, there is the possibility that models might adopt or even amplify these healthcare disparities.

Currently, there is a growing interest in developing healthcare indicators for quality assessment and reimbursement, such as length of stay, thirty-day complication rate, non-routine discharge rate, unplanned readmissions, and patient reported outcome measures. Although these metrics are highly suitable as outcome measures in machine learning models, these algorithms do not have an understanding of good clinical practice, let alone a moral compass. They just aim to optimize the performance according to the metric that is used for analysis, regardless of any other clinical or ethical considerations. It is important, however, to understand that most of these indicators are constructs to measure, objectify, and perhaps quantify clinical care, but do not reflect good practice in itself. For example, improving overall survival is not necessarily desirable if it drastically reduces health related quality of life, and reducing the length of stay is not effective if it comes at a cost of increased thirty-day complication or readmission rate. Besides this unintentional divergence between quality indicators and good clinical practice, models can also be trained for this purpose intentionally. A decision-support tool can be programmed to increase profits for stake holders while ensuring to meet the minimum quality standards. It is therefore imperative that the potential power of such tools is monitored and regulated to ensure they are used for improving patient care above all else. While it is not possible to regulate the internal mechanisms and calculations derived by the algorithm, it will still be possible to monitor the input data, output results, the training datasets used, and the way these models are used to guide clinical care.

## 5. Future Direction

To facilitate a safe and effective implementation of machine learning in the clinical care of glioma patients, we recommend several solutions. As large-scale, high-quality data is the cornerstone of model performance, future research should focus on harmonizing data acquisition parameters, such as the MRI machine settings, pathology specimen preparation protocols, and reporting standards for free text clinical notes. Furthermore, cross-institutional collaboration should be encouraged to gain sufficient training data but also improve external generalizability.

To ensure transparency with regard to the intent and construction of each model, we support the concept of open-source coding as a requirement for the acceptance of manuscripts submitted to journals or for models that are eligible for implementation in clinical care. Making the code available on data repositories, such as GitHub, allows for reproducible research, external validation, and a timely detection and resolution of coding errors.

A better understanding of the promises and pitfalls among clinicians can facilitate a safe and effective deployment of machine learning in clinical practice. By improving the interpretability of constructed models but also the computational knowledge among clinicians, a dependency on “black-box” algorithms can be reduced, and a doctor-versus-machine paradigm can be shifted to a doctor-and-machine paradigm. Several studies that evaluated the combined performance of clinical experts and machine learning models demonstrated a better performance compared to clinicians or models on their own [14]. Furthermore, a better understanding allows clinicians to contribute to model development and timely anticipate deviating performance trends of implemented models, instead of responding to negative patient outcomes further down the line. It is therefore of great importance that a basic level of understanding of machine learning is incorporated into the education and training requirements for physicians.

Although machine learning can produce highly accurate models, clinicians remain responsible for interpreting the clinical and personal implications of these predictions. After all, personalized medicine is not a concatenation of decisions that follow directly from series of accurate predictions. Machine learning-informed decision-making can be very different in two patients even if their predicted outcomes are the same. Machine learning should, therefore, not be considered as a substitute for clinical decision-making, but as a very powerful tool to unlock insight from untapped data sources and an adjunct to clinical decision-making.

## 6. Conclusion

The thin line between treatment effectiveness and patient harms underpins the importance of tailoring clinical management to the individual glioma patient. The use of machine learning provides clinicians with the analytical support for personalizing important treatment decisions. Machine learning, and deep learning in particular, is capable of detecting patterns in large and complex data sets that might not even be detectable or understandable by clinical experts, thereby allowing clinicians to analyze the increasing amount of imaging, genomic, pathology, and free text data that is generated in glioma patients. The astonishing performance, as well as the lack of interpretability, raises ethical concerns in the way clinical decision-making is balanced between man and machines. Future challenges include the assembly of well-curated cross-institutional datasets, improvement of the interpretability of machine learning models, and balancing novel evidence-based decision-making with its associated liability of automated inference. Although artificial intelligence already exceeds clinical expertise in a variety of applications, clinicians remain responsible for interpreting the implications of, and acting upon, each prediction.
